# Adding Emulsified Isoflurane to Cardioplegia Solution Produces Cardiac Protection in a Dog Cardiopulmonary Bypass Model

**DOI:** 10.1038/srep23572

**Published:** 2016-04-28

**Authors:** Han Huang, Cheng Zhou, Jin Liu, Haibo Song, Yan Qiu

**Affiliations:** 1 Department of Anesthesiology, West China Second Hospital, Sichuan University, Chengdu, Sichuan, China; 2 Department of Anesthesiology and Translational Neuroscience Center, West China Hospital of Sichuan University, Chengdu, Sichuan, China

## Abstract

This study investigated whether caridoplegia solution with Emulsified Isoflurane (EI) could improve cardiaoprotection in a dog CPB model of great similarity to clinical settings. Adult dogs were randomly assigned to receive one of the following cardioplegia solutions: St. Thomas with EI (group ST+EI), St. Thomas with 30% Intralipid (group ST+EL) and St. Thomas alone (group ST). The aorta was cross-clamped for two hours followed by reperfusion for another two hours, during which cardiac output was measured and dosages of positive inotropic agent to maintain normal hemodynamics were recorded. Serum level of cardiac troponin I (cTnI) and CK-MB were measured. Deletion of cardiac mitochondrial DNA was examined at the end of reperfusion. Compared with ST, ST+EI decreased the requirement of dopamine support while animals receiving ST+EI had a significantly larger cardiac output. ST+EI reduced post-CPB release of cTnI and CK-MB. Mitochondrial DNA loss was observed in only one of the tested animals from group ST+EI while it was seen in all the tested animals from group ST+EL and ST. Addition of emulsified isoflurane into cardioplegia solution protects against myocardial ischemia reperfusion injury. This protective effect might be mediated by preserving mitochondrial ultrastructure and DNA integrity.

Most open heart surgeries require cardiopulmonary bypass (CPB), so that the heart beat can be safely stopped to provide an operable surgical field. However, the aortic cross-clamping-unclamping causes global myocardial ischemia/reperfusion (I/R) injury. Even with improved perioperative myocardial preservation strategies, postoperative myocardial injury (measured as elevated serum cardiac enzymes such as cardiac troponin I, cTnI and CK-MB) remains an independent risk factor for postoperative adverse outcomes. Bernard *et al*. showed that increased cTnI levels at 24 hours after cardiac surgery were independent predictors of death at 30 days, 1 year, and 3 years. Patients with cTnI levels in the highest quartile were at particular risk^1^. Similarly, Sorin *et al*. demonstrated extensive elevated serum CK-MB level after heart surgery was independently correlated with increased mortality over a three-year period^2^.

Oxidative damage plays an important role in myocardial I/R injury, which leads to impaired mitochondrial function^3^. In aged heart, it is reported that oxidative stress causes damages to mitochondrial DNA (mtDNA) and results in mtDNA deletion^4^. Considering the large amount of free radicals released during myocardial I/R injury^5^, it is expected that the mtDNA deletion would also be observed after CPB.

Emulsified Isoflurane (EI) is an emulsion formulation of isoflurane, which has been demonstrated as a safe intravenous anesthetic in human^6^. With an *in vitro* rat myocardial I/R injury model, we have demonstrated that adding EI into St. Thomas cardioplegia provides additive cardioprotection compared with St. Thomas cardioplegia alone^7^. However, the efficacy of this maneuver still needs validation in animal models much closer to clinical scenarios, such as in a larger animal CPB model. Our previous study has also demonstrated that EI produces myocardial protection by preserving mitochondrial ultrastructure^8^. It is not clear yet whether mtDNA deletion is associated to impaired myocardial function and/or damaged mitochondrial ultrastructure and whether EI could prevent mtDNA deletion caused by CPB.

We therefore hypothesized that St. Thomas cardioplegia solution supplemented with EI would reduce CPB-related myocardial I/R. To test our hypothesis, we performed this animal study to evaluate whether addition of EI into St. Thomas cardioplegia solution could reduce the postoperative release of myocardial injury biomarkers and the requirement for inotropic support agents in dogs undergoing CPB. Meanwhile, we tried to elucidate the underlying mechanisms by focusing on the mitochondrial DNA deletion following myocardial I/R injury.

## Results

### EI improved cardiac functions after CPB

As shown in table 1, before aortic cross-clamping, no significant difference was detected in hemodynamic between the three groups. During the 2-hrs reperfusion, animals in group ST+EI maintained larger cardiac output compared with animals in the other two groups (p < 0.01 v.s. animals in ST+EL or ST). Meanwhile, animals in group ST+EI required less dopamine (3.5 ± 1.4 mcg kg^−1^ min^−1^) to keep systolic blood pressure above 90 mmHg (p < 0.01 v.s. animals in group ST+EL or ST). Group ST+EL also required less post-CPB inotropic support (6.8 ± 1.7 mcg kg^−1^ min^−1^), compared with ST (8.3 ± 2.8 mcg kg^−1^ min^−1^, p < 0.05). There was no difference in heart rate or systolic blood pressure between the three groups.

### EI reduced myocardial enzymes release after CPB

**Figure 1:**
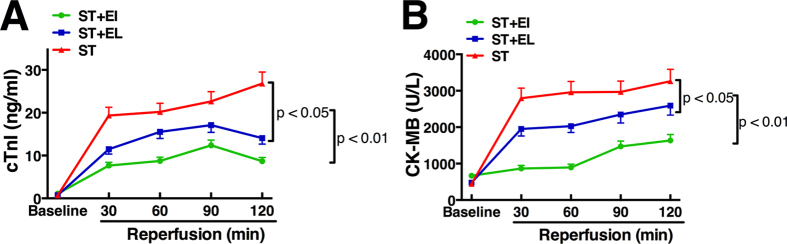
Assessment of myocardial reperfusion injury by cardiac troponin I (cTnI) and CK-MB release measurement. ST+EI significantly reduced the post-CPB release of cTnI **(A)** and CK-MB **(B)** compared with ST (p < 0.01). ST+EL also reduced the release of both markers compared with ST (p < 0.05). EI = Emulsified Isoflurane; EL = Emulsified Intralipid; ST = St. Thomas cardioplegia solution. T bars denote SD (n = 6).

At the end of the 2-hrs reperfusion after CPB, the area under the curve (AUC) for cTnI release was 30 ± 7 units in group ST+EI, which was significantly lower than that in group ST+EL (48 ± 12 units) and group ST (72 ± 11 units), with a p value less than 0.01 as showed in Fig. 1A. Compared with animals group ST, animals in group ST+EL also had a lower release of cTnI (p < 0.05). Similarly, the AUC for CK-MB release during the 2-hrs reperfusion in group ST was 10573 ± 1158 units, which was 4379 ± 629 units in group ST+EI (p < 0.01) and 7858 ± 757 units in group ST+EL (p < 0.05), as showed in Fig. 1B.

**Figure 2:**
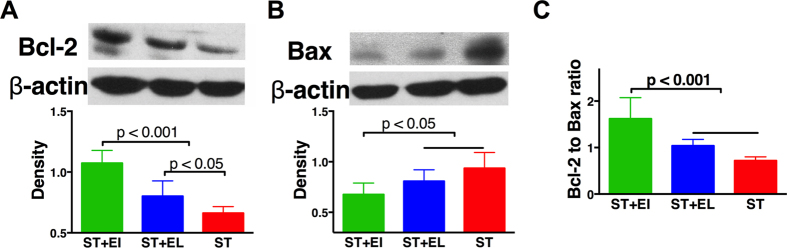
ST+EI inhibits myocardial apoptosis after CPB. **(A)** ST+EI significantly activates anti-apoptotic signaling, reflected as the increased expression of Bcl-2. Compared with ST alone, ST+EL also increases the expression level of Bcl-2. **(B)** ST+EI significantly inhibits pro-apoptotic signaling, reflected as the decreased expression of Bax. **(C)** With activation of anti-apoptotic signaling and inhibition of pro-apoptotic signaling, ST+EI significantly suppresses myocardial apoptosis. EI = Emulsified Isoflurane; EL = Emulsified Intralipid; ST = St. Thomas cardioplegia solution. Unlablled horizontal bar indicates p > 0.05 Data are presented as mean plus SD (n = 6).

Cardiomyocytes apoptosis, was also reduced in animals receiving ST+EI. Compared with ST, ST+EI induced a more-than-2-folds increase in Bcl-2 (anti-apoptotic marker, Fig. 2A) level while a more-than-50% decrease in Bax (pro-apoptotic marker, Fig. 2B) level. These changes lead to a significant activation of anti-apoptotic activity in group ST+EI, reflected as increase in the Bcl-2 to Bax ratio (Fig. 2C). Compared with group ST, the Bcl-2 level was also significantly elevated in group ST+EL, although the differences in Bcl-2-to-Bax ratio did not reach a statistical significance.

### EI attenuated mitochondrial oxidative stress and preserved mitochondrial ultrastructure

**Figure 3:**
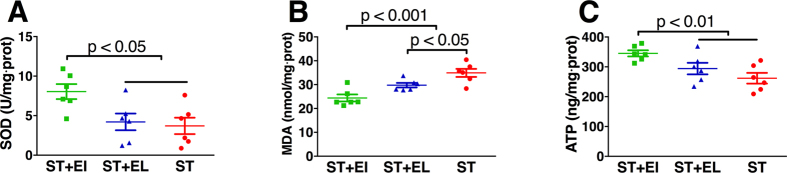
ST+EI attenuates myocardial mitochondrial oxidative stress after CPB. ST+EI significantly increases the level of antioxidant defensive SOD **(A)** while decreases the level of MDA, the marker for oxidative stress. ST+EL also reduces the level of MDA **(B)**. ST+EI preserves the mitochondrial ATP level compared with other two groups **(C)**. EI = Emulsified Isoflurane; EL = Emulsified Intralipid; ST = St. Thomas cardioplegia solution; SOD =  superoxide dismutase; MDA = malondialdehyde. Data are presented as mean plus SD (n = 6).

As showed in Fig. 3, EI significantly activated antioxidant defense by increasing mitochondrial SOD level in group ST+EI (8.0 ± 2.3 IU/mg·prot vs. 4.2 ± 2.6 IU/mg·prot in group ST+EL and 3.7 ± 2.5 IU/mg·prot in group ST, p < 0.05). And the marker of oxidative stress, MDA, was significantly reduced in group ST+EI (24.4 ± 3.6 nmol/mg·prot), compared with that in group ST+EL (29.8 ± 2.3 nmol/mg·prot, p < 0.001) and ST (34.9 ± 4.1 nmol/mg·prot, p < 0.001). It is worth noting that, significant higher lower level of MDA was found in animals receiving ST+EL compared with animals receiving ST (p < 0.01 and p < 0.05, respectively). Mitochondrial ATP was better preserved in animals receiving ST+EI (345.1 ± 25.3 ng/mg·prot), compared with animals receiving ST (261.8 ± 44.2 ng/mg·prot, p < 0.01) or ST+EL (294.3 ± 47.4 ng/mg·prot, p < 0.01).

**Figure 4:**
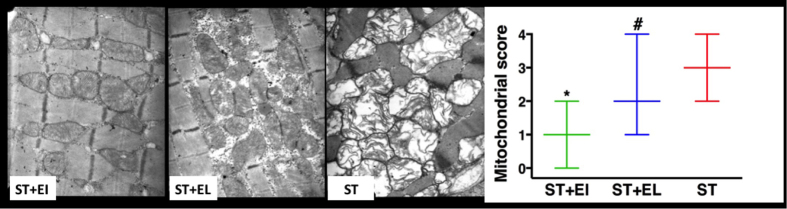
ST+EI preserves myocardial mitochondrial ultrastructure after CPB. Higher mitochondrial scores were observed in animals from ST+EI group and preventative TEM figures were showed (×12,000). EI = Emulsified Isoflurane; EL = Emulsified Intralipid; ST = St. Thomas cardioplegia solution. Data are presented as median and range. *p < 0.05 versus ST+EL or ST; ^#^p < 0.05 versus ST (n = 6).

EI reduced the severity of mitochondria injury, as evidenced by significantly lower mitochondrial injury score in group ST+EI. It remained worth noting that animals in group ST+EL preserved better mitochondrial ultrastructure than animals in group ST, as evidenced by a significantly lower mitochondrial injury score in group ST+EL. Representative TEM figures and mitochondrial ultrastructure morphology scores were presented in Fig. 4.

### EI prevented mtDNA deletion after cardiopulmonary bypass

**Figure 5:**
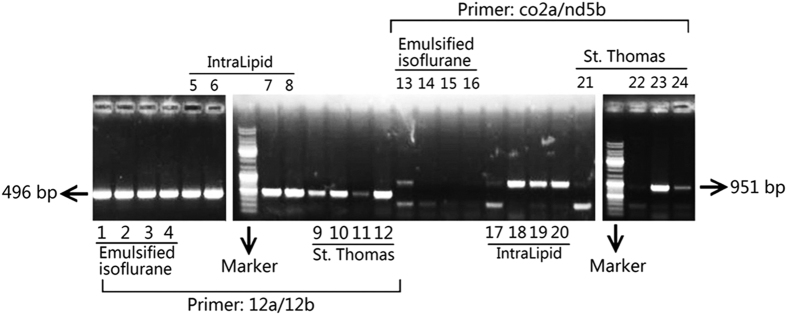
ST+EI prevents myocaridal mitochondrial DNA deletion after CPB. The mitochondrial DNA deletion, reflected as the 951 bp DNA segment, was observed in only one of the four tested animals (Lane 13) from group ST+EI while it was seen in all the four tested animals from group ST+EL and ST. The internal control, the 496 bp band, was seen in each tested animal from all the three groups. EI = Emulsified Isoflurane; EL = Emulsified Intralipid; ST = St. Thomas cardioplegia solution (n = 4).

As shown in Fig. 5, as the internal reference, the 496bp mitochondrial DNA segment was seen in all the lanes. For the detecting primer set, the 951 bp segment was amplified in one out of the four lanes in ST+EI group (lane 13 in Fig. 5), while it could be seen in all the four lanes in ST+EL group (lane 17–20 in Fig. 5) and ST group (lane 21–24 in Fig. 5).

## Discussion

This present study demonstrates that addition of EI into cardioplegia solution is effective in preserving cardiac function and reducing CPB-related myocardial ischemia/reperfusion injury in dogs, which is evidenced by the improved post-CPB hemodynamics, reduced serologic markers for myocardial injury, better preserved mitochondrial ultrastructure and prevention of mitochondrial DNA deletion.

In addition to providing further evidences on the protective effect of EI on myocardial I/R injury, this present study also indicated the potential for using EI as a cardioplegia supplement. Compared with our previous study with a rat Lagendorff model^7^, this study was conducted in a dog CPB model, which is much closer to clinical practices. Plus the satisfactory results from our phase I clinical trial^6^, it is rational for us to expect a promising effect of EI on CPB patients.

Compared with gas isoflurane, using emulsified isoflurane as a cardioplegia supplement had several advantages. Delivery of gas isoflurane into cardioplegia requires a specific circuit including a vaporizer and an oxygenator as described in previous studies^10^, which makes the CPB circuit much more complicated and inconvenient for routine clinical practice. On the contrary, emulsified isoflurane, an intravenous formulation of isoflurane, could be easily added with a syringe as we did in this study.

The authors admitted that blood cardioplegia might also contain volatile anesthetics if the patients received volatile anesthetics for anesthesia. However, the amount of volatile anesthetics in the blood of CPB patient could be very low, which was reported to be less than half of that in the oxygenator inlet gas^11^, since the transfer of volatile anesthetics into the CPB circuit was very slow. With the addition of crystalloid cardioplegia solution, the volatile anesthetics level in the final blood cardioplegia was further decreased, which may not reach the minimal concentration required to induce myocardioprotection^12^. However, the cardioplegia solution containing large amount of isofluane can be easily prepared with EI, as we did in this study. Using EI to produce cardioplegia solution with high isoflurane concentration also avoided the side effects associated with general use of isoflurane at high concentration, such as hypotension.

In line with previous studies^13^,^14^, Intralipid alone produced myocardial protection against myocardial I/R injury, as evidenced by the decreased requirement for inotropic drug, decreased release of MDA and better preserved myocardial mitochondrial ultrastructure. It had been demonstrated that activation of the Akt/GSK pathway was involved in Intralipid-induced myocardial protection^15^. The same Akt/GSK signal pathway was also involved in isoflurane-induced myocardial protection^16^. Therefore, it is not surprising for us to find out that EI produced extra myocardial protection than Intralipid alone in this study. The enhanced cytoprotective action of EI might result from the large interfaces between isoflurane-loaded micelles and lipid rafts may serve as ether-releasing reservoirs and form a cellular microenvironment promoting protection signaling^17^.

As reported before, myocardial I/R injury caused damages in mitochondrial ultrastructure and activation of mitochondria-dependent apoptosis^18^. And by preserving mitochondrial ultrastructure and inhibiting mitochondria-dependent apoptosis, ST+EI produced enhanced cardioprotection. A novel finding from this study was that a 4.8-kb mtDNA deletion was detected in dogs from group ST, but not in the group ST+EI, which suggested that EI prevent this large-scale mtDNA deletion. It is not clear yet how this mtDNA deletion was related to the mitochondrial ultrastructural damage and increased apoptosis. However, the deleted mtDNA contained genes coding proteins for metabolism^19^ and the impaired cardiomyocyte energy supply would definitely result in myocardial injury. Future study should focus on unmasking the relationship between mtDNA deletion and impaired mitochondrial function.

There are several limitations in this study. First, the dose-response curve of EI versus myocardial I/R injury was not investigated; instead, a relatively high isoflurane partial pressure was used. Since the primary goal of this study was to evaluate the potential of EI as a cardioplegia supplement, to find out the minimal effective dose or dose with maximal protection is clearly beyond the scope of this study. And the dose-response curve defined in animals cannot be directly extrapolated into human, so the does-response curve should be studied in human. Second, the anesthesia regimen adopted in this study, the sufentanil and midazolam infusion, is not common. The reason was that we would like to avoid the possible interaction between EI and inhaled anesthetics or propofol, both of which were proved to be cardioprotective as summarized in a very recent review^20^. But in clinical practice, the best anesthesia regimen should be explored to produce maximal cardioprotection in CPB patients.

In summary, addition of EI into cardioplegia produced enhanced cardioprotection in a dog CPB model by preserving mitochondrial ultrastructure, prevention mtDNA deletion and mitochondria-dependent apoptosis. These results imply the potential application of EI as a cardioplegia supplement for maximal cardioprotection during CPB.

## Methods

The study protocol was approved by the Institutional Animal Experimental Ethics Committee of Sichuan University with the ethics approval number of 2008077 (Chengdu, Sichuan, China). All the animals received human care and all the experiment procedures were carried out according to the Guide for the Care and Use of Laboratory Animals published by the US National Institutes of Health (NIH Publication No. 85-23, revised 1996). Male mongrel dogs weighing 12–15 kg were used in this study. Dogs were housed in cages individually with a 12-hrs light-dark cycle.

Emulsified isoflurane (EI) was prepared in our laboratory as previously described^7^. Briefly, liquid isoflurane was dissolved into 30% Intralipid^®^ at the volume ratio of 1:11.5 with an isoflurane concentration of 8% (vol/vol). Liquid isoflurane was purchased from Abbott Pharmaceutical Co. Ltd. of Shanghai (Shanghai, China) while 30% Intralipid^®^ from the Sino-Swed Pharmaceutical Co. Ltd. (Wuxi, Jiangsu, China). St. Thomas cardioplegia solution was prepared as previously reported^7^ (in mM): NaCl, 120; KCl, 16; MgCl_2_, 16.6; CaCl_2_, 1.2; NaHCO_3_, 10; pH = 7.8. And 0.3 mL EI was added into 100 mL St. Thomas cardioplegia solution directly, and the mixture had the isoflurane partial pressure of 2.8% ± 0.1%, which was measured by gas chromatograph. (unpublished data).

### Cardiopulmonary Bypass (CPB) model in Dogs

Dogs underwent well-established CPB procedure as reported from our previous study with minor modifications^9^. The dog received *i.m.* injection of the mixture of ketamine (7 mg kg^−1^) and midazolam (0.2 mg kg^−1^) before entering the operation room. After loss of right reflex, peripheral vein was cannulated, *via* which sufentanil (2 mcg kg^−1^) and propofol (2 mg kg^−1^) were injected. Then, rocuronium (1 mg kg^−1^) was used to facilitate endotracheal intubation and the lungs were ventilated mechanically. Hereafter, anesthesia was maintained by continuous infusion of midazolam and sufentanil, at the initial rate of 0.5 mg kg^−1^ h^−1^ and 0.5 mcg kg^−1^ min^−1^, respectively. The infusion rates were adjusted and muscle relaxant (vercuronium) was supplemented by an anesthetist, who was unaware of animal randomization. Femoral artery was cannulated for invasive blood pressure monitoring. A Swan-Ganz pulmonary artery catheter (Edwards Laboratories, CA, USA) was inserted into the pulmonary artery to measure cardiac output and central venous pressure.

All the surgical procedure was performed by a well-trained cardiac surgeon. The heart was exposed through a midsternal incision. Following infusion of heparin (3 mg kg^−1^), the ascending aorta and the right atrial appendage were cannulated. The CPB circuit was composed of a rolling pump (StÖckert II, Munich, Germany), a membrane oxygenator (1500 ml/min, Kewei Medical Ltd., Guangdong, China) and an arterial filter (Kewei Medical Ltd., Guangdong, China). The CPB was primed with Lactate Ringer’s solution supplemented with 5% sodium bicarbonate (10 ml/l), 20% mannitol (2.5 ml/l), furosemide (0.5–1.0 mg/l), dexamethasone (5 mg/l), heparin (10 mg/l) and 10% potassium chloride (5 ml/l).

Cardiac arrest was initiated with cardioplegia after aortic cross-clamping. At this stage, eighteen dogs were randomly allocated to received one of the following cardioplegia solutions (n = 6): ST+EI (EI was dissolved in St. Thomas cardioplegia solution at the ratio of 0.3 ml EI into 100 ml St. Thomas cardioplegia), ST+EL (30% Intralipid, the solvent of EI, was dissolved in St. Thomas cardioplegia solution at the ratio of 0.3 ml Intralipid into 100 ml St. Thomas cardioplegia) or ST (St. Thomas cardioplegia alone). The volume of cardioplegia soulution was fixed at 15 ml/kg and the infusion was delivered at a temperature of 32 °C in all the animals.

A temperature probe was inserted into the nasal cavity. During the 2-hrs cardiac arrest, the nasal temperature was kept at 32.0 ± 1 °C and during the rest time in this study, the nasal temperature was kept at 37.0 ± 1 °C. The cooling and rewarming were archived with a heat exchanger. Arterial pH was maintained in the range of 7.35–7.45 and arterial partial pressure of carbon dioxide (PaCO_2_) was kept in the range of 35–45 mmHg. The levels of electrolytes were kept within normal range all the time. After the 2-hrs aortic cross-clamping, the aortic clamping was released. Parallel circulation lasted for 30 min before dogs were weaned from the cardiopulmonary bypass. One min before release of the aortic clamp, dopamine infusion was initiated at the rate of 4 mcg kg^−1^ min^−1^. Then dopamine infusion rate was adjusted by the anesthetist, to maintain systolic blood pressure above 90 mmHg at the possible slowest rate. Increment of dopamine infusion rate was 1 mcg kg^−1^ min^−1^ each time, followed by 3-min observation before next adjustment if necessary. Once the systolic blood pressure was higher than 90 mmHg for more than 3 min, the dopamine infusion rate would be decreased by 1 mcg kg^−1^ min^−1^, which would be further decreased till the infusion was completely stopped, as long as the blood pressure was kept above 90 mmHg. Cardiac output was measured before aortic cross-clamping as the baseline, and every 30 min during the 2-hrs reperfusion. At the same time points, blood was sampled for CK-MB and cTnI determination by automatic biochemical analyzer (Hitachi 7600, Hitachi Co., Tokyo, Japan). All the dogs were sacrificed with overdose sodium pentobarbital after the 2-hrs reperfusion, then hearts were harvested.

### Preparation of myocardial mitochondrial fraction

Cardiac mitochondrial samples were prepared by differential centrifugation. Briefly, fresh left ventricle samples were homogenized in buffer A (containing 250 mM sucrose; 10 mM Tris-HCl, pH 7.4; 1 mM EDTA, pH 7–8; 1 mM orthovanadate; 1 mM NaF; 0.3 mM phenylmethylsulfonyl fluoride (PMSF); 5 mcg ml^−1^ each of leupeptin, aprotinin, and pepstatin A) and subjected to serial centrifugations of 1000, 10,000, and 100,000 G. The 1000 G pellet (nuclear fraction) was discarded and the 10,000 G pellet (mitochondrial fraction) was washed in buffer A and re-centrifuged at 100,000G. The final pellet was re-suspended in buffer B (containing 150 mM NaCl; 20 mM Tris-HCl, pH 7.4; 10 mM EDTA, pH 7–8; 1 mM orthovanadate; 1 mM NaF; 0.3 mM PMSF; 0. 5 mcg ml^−1^ pepstatin A; 5 mcg ml^−1^ each of leupeptin and aprotinin; and 1% NP-40) and then subjected to a 21,000 G centrifugation for 10 min. The resultant supernatant was collected as the mitochondrial fraction. All preparation procedures were carried out at 4 °C. The concentration of mitochondrial protein was measured by BCA kits (Pierce Co, NY, USA).

### Measurement of ATP, MDA and SOD

The ATP levels in mitochondrial fraction were measured using reverse-phase high-pressure liquid chromatography (Agilent 1100 Series; Agilent Technologies, CA, USA) with an ODS C18 column (5 μm, 4 × 125 mm; Agilent Technologies).

To determine myocardial oxidative damage and myocardial energetic function, mitochondrial superoxide dismutase (SOD) and malondialdehyde (MDA) levels were measured by commercially available kits (Nanjing Jiancheng Bioengineering Institute, Nanjing, China) following the manufacturer’s instruction.

### Western-blotting analysis of Bcl-2 and Bax proteins

The Bcl-2 and Bax protein level in left ventricle sample was determined by western-blotting. Antibodies for Bcl-2 (sc-492) and Bax (sc-493) were purchased from Santa Cruz Biotechnology, INC., CA, USA. The protein bands were captured with an image processor using the Bio-Rad software (CA, USA) and the intensity of the bands was measured using ImageJ (NIH, USA).

### Transmission electrical microscope (TEM) assessment of the left ventricle

Myocardium sampled from cardiac apex were cut into 1 mm blocks and then fixed in 4% formaldehyde and 1% glutaraldehyde in 0.1 M PBS (pH 7.4) overnight. Then tissue blocks were transferred into 8% (0.2M) sucrose in 0.1 M PBS overnight before post-fixed in 1% osmium tetroxide in 0.1 M PBS for one hour. After dehydrated, tissue blocks were embedded and sectioned. Finally, the sections were stained with uranyl acetate and lead citrate and then observed under transmission electrical microscope (H600-4, Hitachi High-Technologies Corporation, Tokyo, Japan).

Myocardial mitochondrial morphology (at the magnification of X12,000) was evaluated by an experienced researcher unaware of animal randomization. 10 randomly-chosen vision fields were assessed for each animal and the score for each field was calculated from the median score of 10 areas chosen randomly.

The morphology of myocardial mitochondria was evaluated according to our mitochondrial scoring criteria: 0 = mitochondrial ultrastructure is normal; 1 = mitochondrial ultrastructure is intact but the mitochondrial granules are missing; 2 = mitochondria are swelling and mitochondrial matrix is clearing; 3 = mitochondrial cristae are broken and/or mitochondrial matrix is clearing or concentrated; 4 = mitochondrial membranes and mitochondrial matrix are absent.

### Detection of myocardial mitochondrial DNA deletion

Total mtDNA from left ventricle myocytes was isolated using commercially available kit (Biovision, Mountain View, CA, US). Two pairs of primers were designed to determine mtDNA deletion as previously described^4^. Primer set of internal reference was 12a: 5′-TAGATACCCCACTATGCTTAGC-3′, 12b: 5′-TACCTTGTTACGACTTGTCTC-3′. And the detecting primer set for a common 4.8-kb mtDNA deletion was CO2a: 5′-TGGCACATGCAGCGCAAGTAG-3′, NB5d: 5′-GTCTTTGGAGTAGAAACCTGT-3′, which may produce a 951 kb band if the deletion is detected. PCR amplifications were performed in total volume of 50 μl containing: DNA template 200 ng; Taq polymerase 1.25 units (Qiagen, Hilden, Germany); dNTP200 μM; MgCl_2_ 2.5 mM; primer20 pM and 1 × PCR buffer. The PCR procedure consisted of 1 cycle of 95 °C for 2 min; 35 cycles of 94 °C for 30 s, 60 °C for 50 s, and 72 °C for 50 s; and 1 cycle of 72 °C for 5 min. The PCR products were electrophoresed on 1.3% agarose gels and then visualized by ethidium bromide staining. The optical intensity of the band was measured with ImageJ (1.49, NIH). And the band would be regarded as missing if the optical intensity was less than 5% of the control band.

## Statistical analysis

Data were expressed as mean ± standard deviation unless otherwise specified. Hemodynamic data and inotropic support doses were analyzed with repeated-measures analysis of variance followed by a Student-Neumann-Keuls test where appropriate. The areas under the curves (AUC) of myocardial injury biomarkers (CK-MB and cTnI) during the 2-hrs reperfusion were compared by one-way analysis of variance followed by Student-Neumann-Keuls when necessary. Mitochondrial scores were compared via the Kruskal-Wallis test, followed by the Mann-Whitney U test when indicated. A p < 0.05 was considered a statistical significance.

## Additional Information

**How to cite this article**: Huang, H. *et al*. Adding Emulsified Isoflurane to Cardioplegia Solution Produces Cardiac Protection in a Dog Cardiopulmonary Bypass Model. *Sci. Rep.* **6**, 23572; doi: 10.1038/srep23572 (2016).

## Figures and Tables

**Table 1:** Hemodynamics and inotropic support requirement.

	Heart Rate (beat/min)						Systolic Blood Pressure (mmHg)				
	Baseline	Reperfusion					Baseline	Reperfusion			
		30 min	60 min	90 min	120 min			30 min	60 min	90 min	120 min
ST+EI	117 ± 10	145 ± 45	142 ± 29	128 ± 10	134 ± 19	ST+EI	105 ± 23	97 ± 4	93 ± 4	98 ± 6	105 ± 13
ST+EL	130 ± 25	170 ± 24	165 ± 28	154 ± 10	142 ± 22	ST+EL	118 ± 14	94 ± 4	105 ± 12	104 ± 6	106 ± 16
ST	134 ± 19	144 ± 38	135 ± 35	133 ± 33	130 ± 22	ST	125 ± 8	94 ± 4	100 ± 3	104 ± 6	98 ± 5
	**Cardiac Output (L/min)**						**Dopamine (mcg/kg/min)**				
	**Baseline**	**Reperfusion**					**Average**	Reperfusion			
		30 min	60 min	90 min	120 min			30 min	60 min	90 min	120 min
ST+EI	2.69 ± 0.33	2.98 ± 0.71	3.04 ± 0.52	3.16 ± 0.73	3.28 ± 0.73	ST+EI	3.5 ± 1.4	4.0 ± 1.2	3.2 ± 1.4	3.2 ± 1.4	3.2 ± 1.2
ST+EL*	2.15 ± 1.00	2.18 ± 0.44	2.15 ± 0.83	2.22 ± 0.53	2.38 ± 1.19	ST+EL*^#^	6.8 ± 1.7	7.0 ± 1.1	7.2 ± 2.3	6.2 ± 3.4	6.2 ± 3.4
ST*	2.42 ± 0.26	2.09 ± 1.11	2.26 ± 0.71	2.08 ± 0.43	2.50 ± 0.48	ST*	8.3 ± 2.8	7.0 ± 1.7	9.7 ± 3.0	8.5 ± 3.9	6.9 ± 1.6

Data are presented as mean ± SD. *p < 0.01 vs. ST+EI; ^#^p < 0.05 vs. ST. EI = Emulsified Isoflurane; EL =  Emulsified Intralipid; ST = St. Thomas cardioplegia solution (n = 6).
